# A Comparative Transcriptome and Metabolome Combined Analysis Reveals the Key Genes and Their Regulatory Model Responsible for Glucoraphasatin Accumulation in Radish Fleshy Taproots

**DOI:** 10.3390/ijms23062953

**Published:** 2022-03-09

**Authors:** Xiaoman Li, Peng Wang, Jinglei Wang, Haiping Wang, Tongjin Liu, Xiaohui Zhang, Jiangping Song, Wenlong Yang, Chunhui Wu, Haohui Yang, Liwang Liu, Xixiang Li

**Affiliations:** 1 National Key Laboratory of Crop Genetics and Germplasm Enhancement, Key Laboratory of Horticultural Crop Biology and Genetic Improvement (East China) of Ministry of Agriculture and Rural Affairs, College of Horticulture, Nanjing Agricultural University, Nanjing 210095, China; lxm911001@163.com; 2 Key Laboratory of Biology and Genetic Improvement of Horticultural Crops, Institute of Vegetables and Flowers, Chinese Academy of Agricultural Sciences, Ministry of Agriculture and Rural Affairs, Beijing 100081, China; 82101172227@caas.cn (P.W.); syauwjl@163.com (J.W.); wanghaiping@caas.cn (H.W.); tongjinliu@163.com (T.L.); zhangxiaohui01@caas.cn (X.Z.); songjiangping@caas.cn (J.S.); yangwenlong@caas.cn (W.Y.); wchd163@163.com (C.W.); yanghaohui127@163.com (H.Y.); 3 Institute of Vegetables Research, Zhejiang Academy of Agricultural Sciences, Hangzhou 310021, China; 4 Jinling Institute of Technology, College of Horticulture, Nanjing 210038, China

**Keywords:** glucosinolate profiles, RNA-seq, *RsMYB28*, key genes, regulatory network, radish fleshy taproots, genotypes

## Abstract

Radish (*Raphanus sativus* L.) is rich in specific glucosinolates (GSLs), which benefit human health and special flavor formation. Although the basic GSLs metabolic pathway in Brassicaceae plants is clear, the regulating mechanism for specific glucosinolates content in radish fleshy taproots is not well understood. In this study, we discovered that there was a significant difference in the GSLs profiles and the content of various GSLs components. Glucoraphasatin (GRH) is the most predominant GSL in radish taproots of different genotypes as assessed by HPLC analysis. Further, we compared the taproot transcriptomes of three radish genotypes with high and low GSLs content by employing RNA-seq. Totally, we identified forty-one differentially expressed genes related to GSLs metabolism. Among them, thirteen genes (*RsBCAT4*, *RsIPMDH1*, *RsMAM1a*, *RsMAM1b*, *RsCYP79F1*, *RsGSTF9*, *RsGGP1*, *RsSUR1*, *RsUGT74C1*, *RsST5b*, *RsAPK1*, *RsGSL-OH*, and *RsMYB28*) were significantly higher co-expressed in the high content genotypes than in low content genotype. Notably, correlation analysis indicated that the expression level of *RsMYB28*, as an R2R3 transcription factor directly regulating aliphatic glucosinolate biosynthesis, was positively correlated with the GRH content. Co-expression network showed that *RsMYB28* probably positively regulated the expression of the above genes, particularly *RsSUR1*, and consequently the synthesis of GRH. Moreover, the molecular mechanism of the accumulation of this 4-carbon (4C) GSL in radish taproots was explored. This study provides new perspectives on the GSLs accumulation mechanism and genetic improvements in radish taproots.

## 1. Introduction

Glucosinolates (GSLs) are a large class of important sulfur-rich secondary metabolites of Brassicaceae plants [1,2]. Based on the precursor amino acids and R groups modification, GSLs are divided into aliphatic, aromatic, and indole groups [1,2,3]. So far, more than 200 types of GSLs have been found in the cruciferous family [4]. Different *Brassicaceae* plants have diverse GSLs, such as 4-methylthiobutyl as the main GSL in *A. thaliana* root [5]; gluconapin is mainly present in *B. rapa* [6] and Chinese kale [7]; glucoraphanin (GRA) is present in broccoli seed [8], and glucoerucin exists in cabbage (*Brassica oleracea*) [9]. GSLs and myrosinases (b-thioglycoside glucose hydrolase, EC 3.2.3.1) are usually stored in different cell compartments. After tissue damage, GSLs are released from the vacuole and rapidly hydrolyzed by myrosinases to glucose and thiohydroxymate O-sulfonate, and the latter is unstable and spontaneously becomes isothiocyanate (ITCs), thiocyanate, nitrile, acetonitrile, or oxazolidine-2-thioketone [6,7]. GSLs and their degradation products have significant biological activities, such as plant disease resistance and inhibition of human cancer cells, and they are also special flavor substances [1,10,11,12], although some of them have side effects, such as sinigrin [13] and cyanogenic glucosides [14], which may influence the normal function of the thyroid, and Epithionitriles, nitriles, and sulforaphane nitrile, which have less opportunity to be converted into beneficial products [15,16].

The aliphatic GSLs biosynthetic pathway in *Arabidopsis thaliana* has been elucidated and includes three stages: side chain elongation of amino acids, development of the core structure, and secondary side chain modifications [2,17]. The GSLs biosynthetic pathway is involved in many genes and transcription factors (TFs). At the stage of GSLs chain elongation, *BCAT3* and *BCAT4* perform the deamination and transamination of the Met chain elongation cycle in the formation of aliphatic GSLs [18,19,20]. *MAM1* and *IPMDH1* catalyze the elongation of the side chain length of aliphatic GSLs [21,22]. With regard to the biosynthesis of core GSLs structure, firstly, precursor amino acids are converted to aldoximes by *CYP79F1*/*CYP79F2* [23,24], and aldoximes are then oxidized to activated compounds (either nitrile oxides or aci-nitro compounds) by *CYP83A1* [25]. After conjugation of the activated aldoximes to a sulfur donor, which can occur nonenzymatically, the produced S-alkylthiohydroximates are converted to thiohydroximates by the C-S lyase SUR1 [26]. Thiohydroximates are then S-glucosylated by glucosyltransferases of the *UGT74* family to form desulfo-GSLs [27]. Glucosylation gives rise to desulfo-GSLs, which are ultimately sulfated by sulfotransferases (SOT16, 17, and 18) to form GSLs [28], and then *FMOGS-OX5*, *AOP1*, *GSL-OH*, *CYP81F2*, and *CYP81F4* genes are involved in the next steps of secondary modification. Although this basic GSL pathway is shared by other Brassicaceae plants, there are some modifications in their GSL metabolic pathways responsible for diverse GSLs and catabolites in different plants [29,30].

In *A. thaliana*, *MYB34*, *MYB51*, *MYB122*, *MYB28*, *MYB29*, and *MYB76* are six TFs that have been identified as regulators of GSLs biosynthesis. The former three MYBs regulate the biosynthesis of indole GSLs, whereas the latter three MYBs regulate the aliphatic biosynthesis of GSLs [31,32]. *MYB28* as main regulator is followed by *MYB29*, while *MYB76* plays a minor role in the aliphatic GSLs formation [33]. *BoaMYB28* has the potential to alter the aliphatic GSLs content in *Brassica oleracea* at the genetic level [34]. *MYB28* is also a regulator of Met-derived GSLs biosynthesis in *Brassica juncea* [35,36]. *BoMYB29* enhances the expression of aliphatic GSLs biosynthetic genes and the accumulation of aliphatic GSLs in *Brassica oleracea* [37]. *MYB28* genes have been detected at three QTLs associated with the GSLs content in rapeseed [38]. Besides, *IQD1* integrates intracellular Ca^2+^ signals to fine-tune GSLs accumulation in response to biotic challenge [39]. *OBP2* is also part of the regulatory network that regulates GSLs biosynthesis in *A. thaliana* [40]. It is obvious that different TFshave different roles in different plants.

Radish (*Raphanus sativus* L., 2n = 2x = 18) is a member of the plant family Brassicaceae and is also a historically important cultivated crop all over the word [41]. It was reported that there were different types of main GSLs in *R. sativus* [9,42,43,44,45,46]. Among them, glucoraphasatin and glucoraphenin, which belong to the aliphatic group, are the two predominant types [3,42,47]. Sulforaphene, produced by the breakdown of glucoraphenin, obtained from hydrolysis of glucoraphasatin, strongly influences the taste of *R. sativus* roots [42] and has potential antioxidative and antimutagenic [48], anti-inflammatory [49], cardiovascular protection, and anticarcinogenic activities [50]. Compared with other Brassicaceae plants, the GSLs metabolic pathway in radish is specific in some respects with regard to the synthesis of particular components, although many similarities among the pathways exist [29]. *RsFMOs* are responsible for converting GRH into glucoraphenin (GRE). With regard to GSLs accumulation in radish taproot, one study reported that three large QTL intervals may be related to the content of aliphatic GSLs in radish fleshy roots [51]. The *FMOGS-OX2* gene expressed differentially in radish taproots and siliques produced different aliphatic GSLs components—that is, GRH in radish taproots and GRE in seeds, respectively [43,52]. *RsGRS1* participates in the dehydrogenation reaction to generate unsaturated 4-carbon (4C) GSLs from glucoerucin or glucoraphanin to obtain GRH or GRE, respectively [9]. Kang et al. [53] analyzed the GSLs content of 59 accessions of radish germplasm and selected out 5 radish accessions with a significant difference in total GSL content for transcriptome analysis. They identified 13 differentially expressed genes (DEGs) in the aliphatic GSL biosynthesis pathway and one differentially expressed transcription factor *MYB29* (*RSG00789*) among these five roots. However, it is noteworthy that all the DEGs showed very low expression without any transition in the fleshy taproots of different genotypes with low and moderate GRH concentrations. In addition, they did not elucidate both the regulation relationship among these DEGs and the relationship between GRH content and *MYB29*. Therefore, the key genes and regulation mechanism for the specific GSLs accumulation is still poorly understood in the fleshy taproots of different radish genotypes. In this study, we selected out three radish genotypes with marked differences in total GSLs content from a previous study [54]. We used HPLC to analyze the GSL profiles and their component contents of the fleshy taproots of these three genotypes. At the same time, we employed a transcriptome method to compare the taproot gene expression profiles of these three genotypes, identified the crucial gene-related GSL biosynthesis, and constructed a metabolic network for the accumulation of GSLs in radish fleshy taproots by combining co-expression analysis and qRT–PCR verification of DEGs and correlation analysis of key gene expressions and major metabolites content. These results provide insights into the relationship among the expression of regulatory factors of GSLs biosynthesis and the key genes related in GSLs synthesis in radish fleshy roots. This study will be beneficial for an in-depth understanding of the mechanism regulating GSLs accumulation and for the molecular genetic improvement in the GSLs content in radish.

## 2. Results

### 2.1. GSLs Profiles and Content of Various GSL Components in the Radish Taproots of Different Genotypes

**Figure 1:**
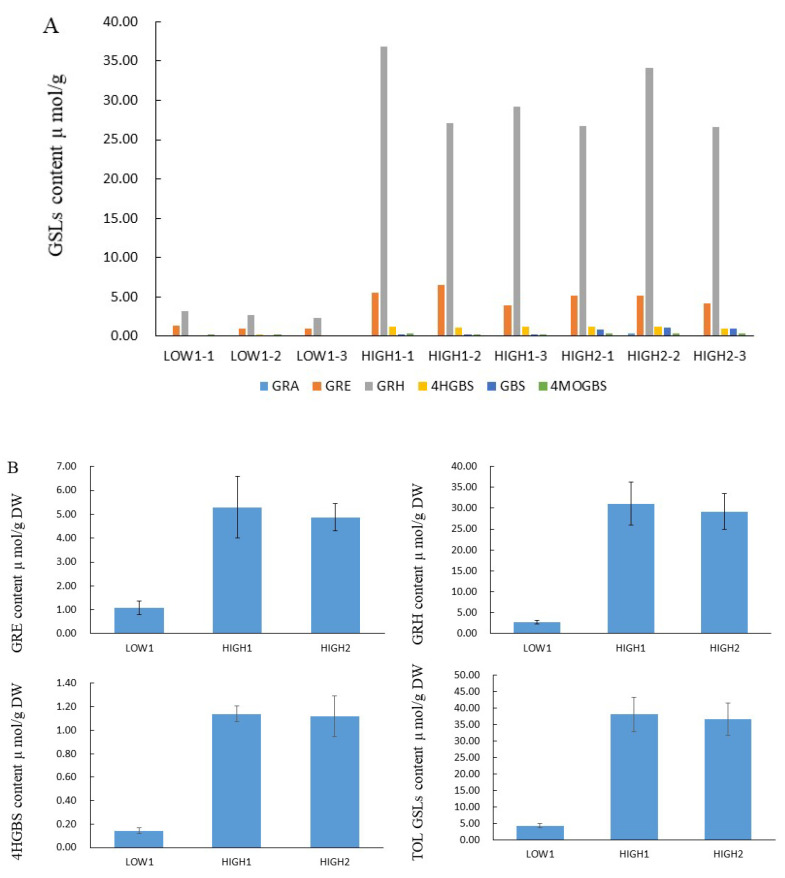
GSL profiles and content of various GSLs in the fleshy taproots of LOW1, HIGH1, and HIGH2 genotypes. (**A**): The *x* axis shows the GSL profiles of different biological replicates of LOW1, HIGH1, and HIGH2 fleshy taproots. The different color bars indicate GRA, GRE, GRH, 4-hydroxyglucobrassicin (4HGBS), glucobrassicin (GBS), and 4MeO-glucobrassicin (4MOGBS). The *y* axis shows the content of individual GSLs in LOW1, HIGH1, and HIGH2 fleshy taproots. (**B**): The main GSLs content in radish taproot.

We analyzed GSLs components and their content by using the HPLC method (Figure 1, Table S2). Totally, we identified out six GSL components, which included three aliphatic glucosinolates (GRA, GRE, and GRH) and three indolyl glucosinolates (4-hydroxyglucobrassicin: 4HGBS, glucobrassicin: GBS, and 4-Methoxyglucobrassicin: 4MOGBS). The aliphatic GSLs were prominent, and the proportion of aliphatic GSLs accounting for the total GSLs content was 89.67% in LOW1, 98.53% in HIGH1, and 93.31% in HIGH2. The GRH as the most predominant component had an average value of 2.74, 31.04, and 29.14 μmol/g dry weight (DW) in LOW1, HIGH1, and HIGH2, respectively. The highest values of GRH in HIGH1 was almost 13 times that of the lowest value in LOW1. As for GRE, as the component of second highest content, its content in HIGH1 was almost 4-fold higher than that in LOW1.

### 2.2. Transcriptome Analysis of Fleshy Taproots of Three Different Radish Varieties

To clarify the differences in transcription levels between above three radish varieties, nine cDNA libraries (HIGH1, HIGH2, and LOW1; three replicates of each variety) were constructed using total RNA extracted from the fleshy taproots (Table S3). After removing low-quality reads, the average number of reads per library was higher than 44 million. The Q20 percentages (sequencing error rates lower than 1%) ranged from 97.84% to 98.02%, and the GC percentages ranged from 47.37% to 47.70%. After alignment with the radish reference genome [55] and subsequent analysis using TopHat2, 33,265,682 (70.72%), 33,349,417 (71.10%), 33,048,869 (70.45%), 31,011,460 (66.00%), 31,065,284 (66.04%), 31,005,222 (65.90%), 31,582,144 (70.85%), 33,257,097 (71.20%), and 32,740,986 (70.40%) reads in nine libraries were mapped to the radish reference genome, respectively. Among these, 67.43%, 67.52%, 67.06%, 62.84%, 63.05%, 62.61%, 67.08%, 67.99%, and 67.14% were mapped uniquely to one location. The above-mentioned quality analysis showed that the data were reliable and sufficient.

**Figure 2:**
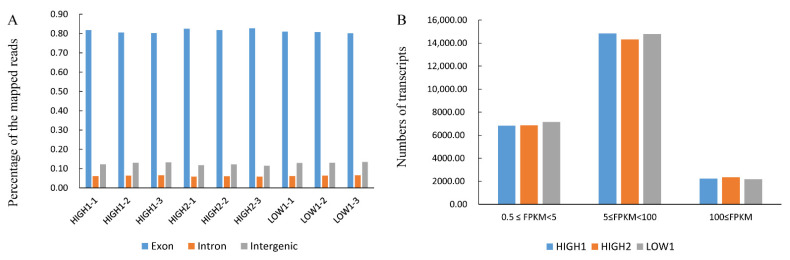
Transcriptome analysis of fleshy taproots of three radish genotypes. (**A**) Percentages of the mapped reads distributed in different sites (intron, intergenic, and exon) of genes in HIGH1, HIGH2, and LOW1. The *x* axis represents three replicates of the sequenced varieties—namely, HIGH1, HIGH2, and LOW1. The *y* axis shows the percentage of mapped reads distributed in different sites of genes. (**B**) Numbers of transcripts with different FPKM values in the roots of the three cultivars. The *x* axis represents the sequenced genotypes—namely, LOW1, HIGH1 and HIGH2. The *y* axis shows the numbers of transcripts. The different colors represent the different FPKM values.

Figure 2A and Table S4 show that more than 80% of the mapped reads were in exons. The number of transcripts identified in each sample is expressed in FPKMs and is shown in Figure 2B and Table S5. Genes with normalized read numbers lower than 0.5 FPKM were removed from the analysis. In total, 23,903, 23,522 and 24,077 transcripts were found to be expressed in HIGH1, HIGH2, and LOW1, respectively. Approximately 29% of the expressed genes were in the range of 0.5 to 5 FPKM, and 62% of the expressed genes were in the range of 5 to 100 FPKM (Figure 2B, Table S5). The comparison of HIGH1 and HIGH2 with LOW1 identified 3936 (2105 upregulated and 1831 downregulated) and 5505 (2651 upregulated and 2854 downregulated) DEGs, respectively. These results indicate that the majority of the genes were differentially expressed in fleshy taproots of different varieties at the mature stage.

### 2.3. Expression of Genes Related to GSLs Biosynthesis and Degradation in Fleshy Taproots of Three Radish Varieties

**Figure 3:**
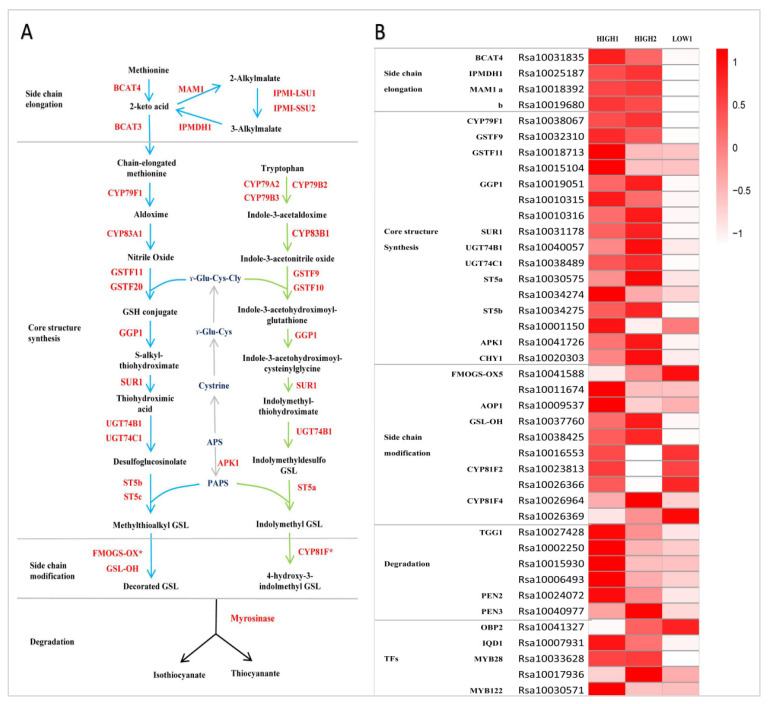
GSLs pathway and its expression pattern in radish fleshy taproots. (**A**) GSLs pathway map. (**B**) Heatmap showing the expression level of genes encoding enzymes and regulators in the GSLs pathway. The color code indicates the differential expression values, and the scale on the right represents the gene expression levels based on the log2 values of reads per kilobase of exon model per million mapped reads (FPKM). BCAT, branched-chain amino acid aminotransferase; MAM, methylthioalkylmalate synthase; IPMI-LSU1, isopropylmalate isomerase large subunit 1; IPMI-SSU2, isopropylmalate isomerase small subunit 2; IPMDH, isopropylmalate dehydrogenase; CYP, cytochrome P450; GSTF, glutathione S-transferase; GGP, gamma-glutamyl peptidase; SUR, C-S lyase SUPERROOT; UGT, UDP-glucosyltransferase; ST, desulfoglucosinolate sulfotransferase; FMO-GSOX, flavin-monooxygenase glucosinolate S-oxygenase; AOP, alkenyl hydroxalkyl producing; GSL-OH, Fe (II)-dependent oxygenase superfamily protein; ESP, epithiospecifier protein; APK, adenylyl sulfate kinase; GSH, gamma-glutamylcysteine synthetase; APS, adenosine-5′-phosphosulfate; PAPS, 3′-phosphoadenosine-5′-phosphosulfate; OBP and IQD, DOF zinc finger proteins; and MYB, MYB domain protein.

**Figure 4:**
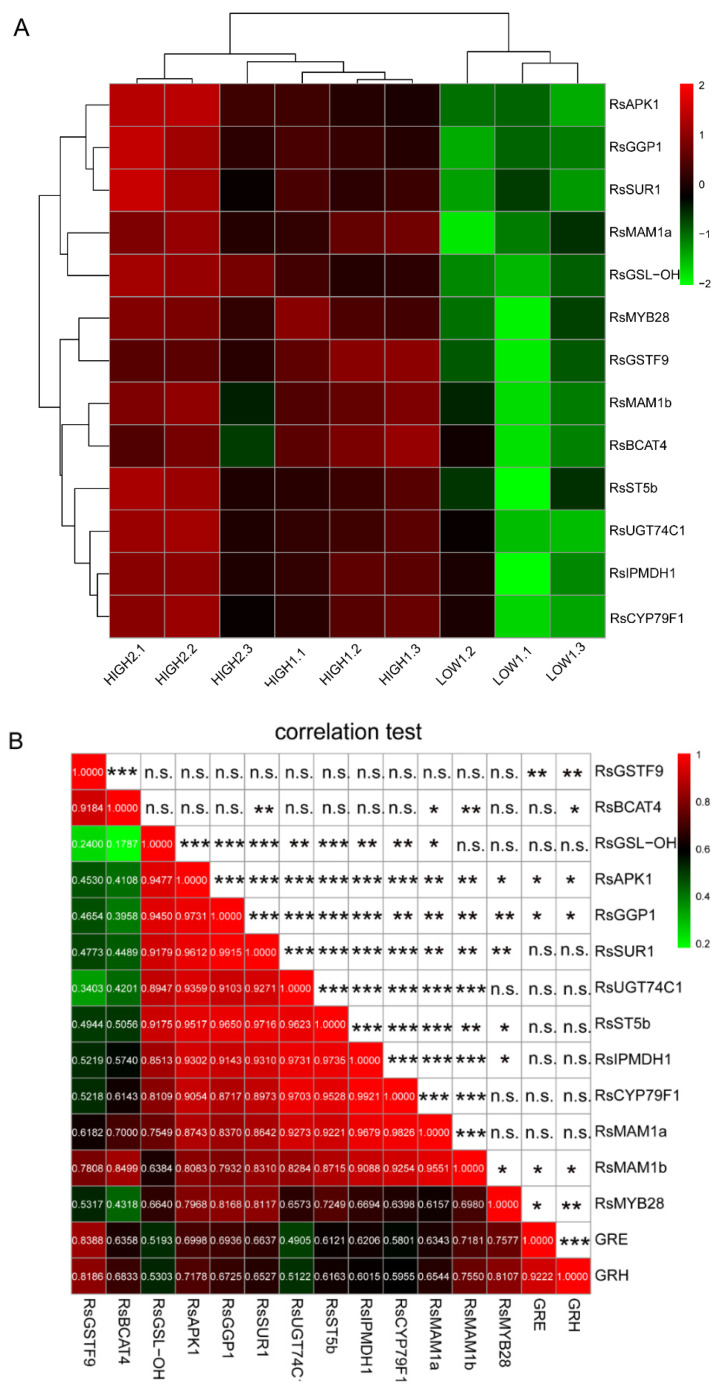
Differential expression of key GSLs pathway-related genes and their correlations in different radish samples. (**A**) Heatmap visualization of the relative gene expression levels in different radish taproots. The legend shows the up- and downregulation expression pattern that was maintained in each sample. (**B**) The Pearson’s coefficient (r) and correlation significance (asterisks) are elucidated in a symmetric matrix prepared using the same variables as in A, with the addition of the GRH and GRE content. The location of the variables in the heatmap are based on the results of hierarchical clustering; the negative and positive correlations are shown in green and red squares, respectively, and the r values are shown in the squares. *, **, and *** indicate significance at *p* ≤ 0.05, *p* ≤ 0.01, and *p* ≤ 0.001, respectively; n.s., not significant.

Firstly, we identified the radish whole genome genes involved in the GSLs metabolic pathway by referring to the GSLs biosynthetic pathway in Arabidopsis. The method and criteria referred to reference [29]. Secondly, based on the transcriptome data, we selected out the DEGs and made the heatmap by comparing the expression of all the identified genes based on the FC and FDR. According to the expression of these genes being involved in the three independent stages of GSLs biosynthesis in three varieties, we identified four DEGs related to side chain elongation, including branched-chain aminotransferase 4 (*BCAT4*), isoropylmalate dehydrogenase (*IPMDH1*), and methylthioalkylmalate synthase (*MAM1*). The expression levels of *RsBCAT4*, *RsIPMDH1*, *RsMAM1a*, and *RsMAM1b* were significantly higher in HIGH1 and HIGH2 than in LOW1 (Figure 3B and Figure 4A).

Totally, we identified sixteen genes functioning in GSL core structure formation in three radish taproot samples (Figure 3B). Among these genes, there were seven DEGs (*RsCYP79F1*, *RsGSTF9*, one copy of *RsGGP1* (*Rsa10019051*), *RsSUR1*, *RsUGT74C1*, one copy of *RsST5b* (*Rsa10034275*), and *RsAPK1*) that showed extremely markedly and repeatedly higher expression in HIGH1 and HIGH2 compared with LOW1 (Figure 3B and Figure 4A); however, *CYP83A1* was not detected in the three cultivars. Among the genes involved in side chain modification, only two copies of *RsGSL-OH* were significantly differentially expressed among three radish genotypes. The *GSL-OH* locus is responsible for biosynthesis of the hydroxylated alkenyl GSLs 2-hydroxybut-3-enyl GSLs, whose production is dependent on the presence of both *AOP2* and *MAM1* [56].

In Arabidopsis, *TGG1*, *TGG2*, *PEN2*, and *PEN3* are four myrosinase genes involved in the degradation of GSLs, and *TGG1* and *TGG2* are well known as important myrosinases [57]. Among these, *PEN2* and *PEN3* cleave indole GSLs. Aliphatic GSLs cannot be degraded in the double mutant, whereas indole GSLs can be reduced slightly in tgg1tgg2, which indicates that *TGG1* and *TGG2* mainly degrade aliphatic GSLs and exert only slight effects on indole GSLs. However, none of these genes were identified as DEGs between the high- and low-GSLs genotypes.

### 2.4. Performance of Transcription Factors Related to GSLs Biosynthesis in the Fleshy Taproots of Three Radish Varieties

In *A. thaliana*, *MYB28*, *MYB29*, and *MYB76* are commonly defined as TFs in the biosynthesis of aliphatic GSLs, and these TFs can specifically activate aliphatic GSLs biosynthesis-related genes such as *MAM3*, *CYP79F1*, and *CYP83A1* [30,58]. In contrast, *MYB34*, *MYB51*, and *MYB122* exclusively transactivate the promoters of *TSB1*, *CYP79B2*, and *CYP79B3*, which are involved in the indole GSLs biosynthetic pathway [59]. In this study, the expression of five genes (*Rsa10041327*, *Rsa10007931*, *Rsa10033628*, *Rsa10017936*, and *Rsa10030571*) were detected and classified as TFs related to GSLs biosynthesis in the expression profiles of radish taproots (Figure 3B). Notably, we noticed that *MYB28* had three copies in radish, which were *Rsa10033628*, *Rsa10019636*, and *Rsa10017936*. Among these three genes, the expression of one copy of *MYB28* homologs (*Rsa10033628*) was significantly higher in HIGH1 and HIGH2 than in LOW1. Furthermore, IQD1 expression was significantly higher in HIGH1 and HIGH2 than in LOW1, whereas other TFs exhibited no significant differences among the three radish varieties.

### 2.5. Establishment of a Co-Expression Network of the Key Genes for GSLs Biosynthesis in Radish Fleshy Taproots

**Figure 5:**
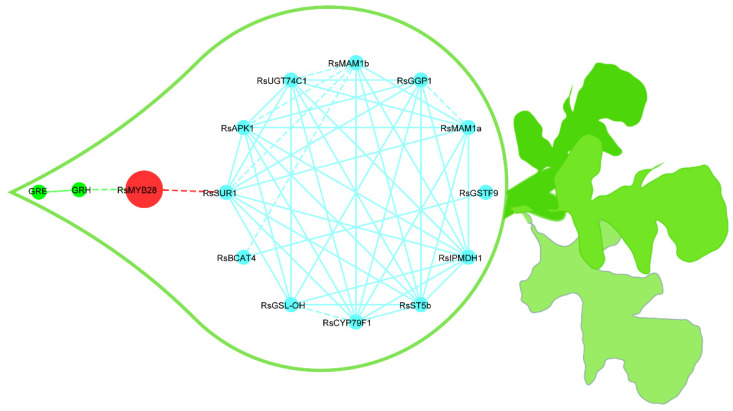
Putative metabolic network for the synthesis of GSLs in radish fleshy taproots. The blue lines represent the relationships among GSLs biosynthesis genes; the red lines represent the relationships between *RsMYB28* and GSLs biosynthesis genes; the green lines represent the relationships between genes and GSLs. The correlation strength according to the r coefficient ranges was used to determine whether the lines are shown as solid or dashed (dashed: 0.7 ≤ r ≤ 0.85; solid: r > 0.85). The red nodes represent transcription factors, the blue nodes indicate GSLs biosynthesis genes, and the green nodes are GSLs.

Based on the differential expression analysis of genes related to the synthesis of GSLs, 13 significant DEGs were found among the three varieties (Figure 4A). After conducting correlation analyses among these key genes and the principal GSLs, we found that the *RsMYB28* transcription was extremely significantly positively correlated with the GRH content. Furthermore, *RsMYB28* was significantly positively associated with *RsSUR1*, which can convert S-alkylthiohydroximates to thiohydroximates during formation of the GSLs core structure (Figure 4B). The 12 key DEGs on the circle (Figure 5) showing distinct expression-based correlation strengths are involved in the formation of two types of GSLs, respectively: aliphatic GSLs, which can be formed by the co-expression of 11 key unigenes in GSLs biosynthesis, and indole GSLs, which are affected by two unigenes, *RsBCAT4* and *RsGSTF9.* No significant correlations were found between other GSLs biosynthesis genes and the corresponding GSLs components. The analyses allowed depiction of a putative gene/metabolite network (Figure 5), which encompassed TFs, *RsMYB28*, 12 GSLs biosynthesis genes, GRH, and GRE. This model will aid further study of the relationship of the candidate genes and GSL accumulation in radish taproots.

### 2.6. qRT–PCR Analysis of DEGs in Three Different Radish Cultivars

**Figure 6:**
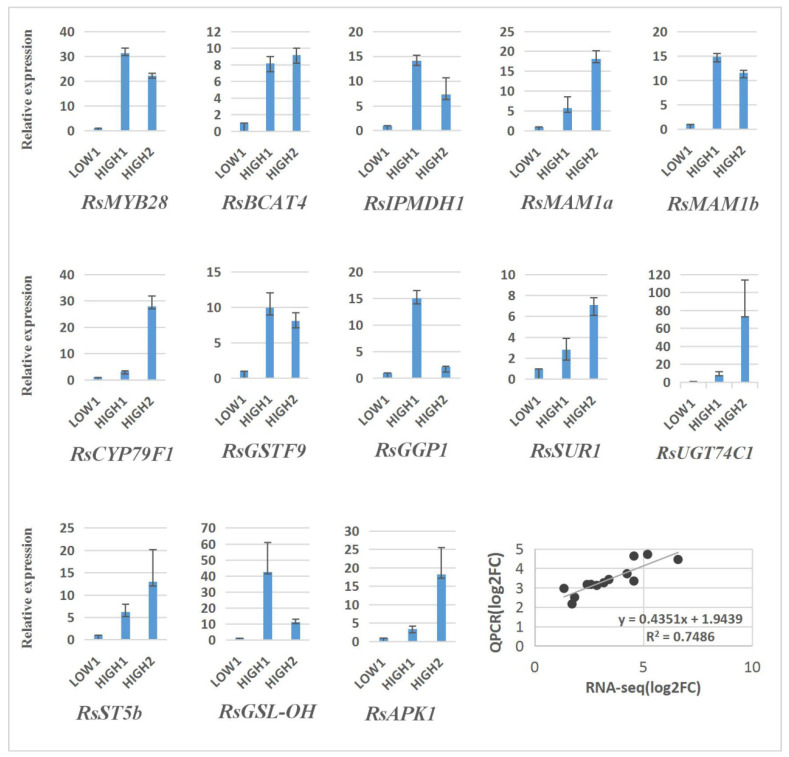
Validation of the expression of 13 differentially expressed genes in LOW1, HIGH1, and HIGH2 fleshy taproots by qRT–PCR. The *x* axis represents the three cultivar taproots. The *y* axis shows the relative expression of one specific gene to the reference gene *RsGAPDH*, and the expression of genes in LOW1 was set to “1” as a control. The last panel shows the results from the RNA-seq/qRT–PCR correlation analysis (bottom right) expressed as log2-fold changes in the low-GSLs vs. high-GSLs genotypes; a significant positive correlation was found between the fold changes in expression, as determined by Pearson’s correlation (R^2^ = 0.75; *p* < 0.001).

In order to verify the reliability of the transcriptome analysis, we validated the sequencing data for 13 unigenes by qRT–PCR, and the results show a similar expression pattern, with the genes exhibiting higher expression levels in HIGH1 and HIGH2 than in LOW1 (Figure 6).

## 3. Discussion

### 3.1. The Content of GRH as the Main GSLs Component in Radish Taproot Is Significantly Different among Different Genotypes

Through HPLC analysis, we found that the GSLs profiles were similar and that GRH was the main component among the six types of GSLs in the radish taproot, followed by GRE. The total GSLs content and components content were significantly different among the three radish genotypes. Additionally, the GRH values in genotype HIGH1 were almost 13 times as high as those in genotype LOW1. Kang et al. [53] found six aliphatic and four indole GSLs in 59 radish accessions and significant difference in total GSLs content and GRH content among different genotypes, although it was puzzling that GRH content was higher than the total GSLs content in RA157-74 and in IT119238-8. Kim et.al [46] detected 13 types of GSLs in radish fleshy taproot skin and flesh and found that the highest total GSLs was 168.44 μmol/g DW in radish skin and 71.09 μmol/g DW in radish flesh. Maldini et al. [42] identified and quantified GSLs in different parts of wild radish and showed that GRH (56 mg 100 g^−1^ FW) was followed by GRE. Ishida et al. [42] analyzed GSLs content in 28 cultivated radishes and found that the GSLs content ranged from 8.6 μmol/g to 137.7 μmol/g and that the predominant GSLs were 4-methylthio-3-butenyl GSLs. Yi et al. [45] analyzed 71 radish accessions and also proved that GRH was dominant (84.5% of total GSLs) in radish root. The types and content of glucosinolates detected in different studies were somewhat different, which may be due to different materials, the precision of analytical equipment, and experimental conditions. In general condition, the types and content of glucosinolates detected in a batch of materials under the controlled condition of a same study were credible and comparable. In general, GRE accounts for 70–95% of total GSLs in seeds, while GRH accounts for 90% of the total GSLs in radish taproots [29,42], differing from what is found in *Brassica* species [60]. Therefore, the radish genotypes with great differences in GSLs content as the model for studying the GRH biosynthesis and metabolic mechanism are valuable.

### 3.2. RsMYB28 Regulatory Model Responsible for GSLs Accumulation in Radish Fleshy Taproots

The expression of *RsMYB28* (*Rsa10033628*) was significantly correlated with the GRH content in radish fleshy taproots (Figure 4B and Figure 5). Meanwhile, *Rsa10033628* was co-expressed with 12 important DEGs related to GSL biosynthesis. It could be inferred that *RsMYB28* was a key regulatory factor responsible for GSLs accumulation in radish fleshy taproots. Kang et al. deduced that it is *MYB29* not *MYB28* that is regulated in GRH biosynthesis in radish root only based on its co-expression with 13 aliphatic GSL metabolic DEGs [53], which are not totally identical to the 12 aliphatic GSL biosynthesis DEGs we identified in the present study. It was a pity that they did not actually analyze the relationship among GRH content, *MYB29*, and GSL biosynthesis genes. The differences in results may be due to different genotypes in these two different studies, which needs to be confirmed by further experiments. However, in *A. thaliana*, *MYB28* is commonly defined as the main transcriptional regulator in the biosynthesis of aliphatic GSLs [30,33,61]. In *B. juncea*, four homologous genes encoding *MYB28* were found to participate in the regulation of aliphatic GSLs biosynthesis [36], and *BoaMYB28* has the potential to alter the aliphatic GSLs content in *Brassica oleracea* at the genetic level [34]. Our investigation confirmed that *RsMYB28* may also be the key gene regulating the content of aliphatic GRH, which is the most abundant GSL in radish fleshy taproots in its own way of cooperating with other genes in GSLs biosynthesis.

Most of the genes related to aliphatic GSLs are regulated by *MYB28* and *MYB29* [62]. A previous study showed that *BrAOP2* and *BrGSL-OH* are negatively and positively regulated in *BrMYB28.1*-overexpressing lines by all three *BrMYB28s*, which indicates that the regulatory mechanism of GSLs biosynthesis in *B. rapa* differs from that in *A. thaliana* [63]. Our results show that *RsMYB28* exhibited a higher significance of expression correlation and co-expression trend with RsSUR1, which converts S-alkyl-thiohydroximates to thiohydroximates (Figure 4B and Figure 5). Therefore, we speculated that *RsMYB28* may promote the accumulation of GSLs in radish fleshy taproots by regulating the expression of *RsSUR1* and another eleven co-expressing DEGs that may be the key genes involved in GSLs biosynthesis.

### 3.3. Key Gene of GSLs Biosynthesis in Radish Fleshy Taproots

Similar key DEGs related to GSL biosynthesis were detected in our study and Kang’s study [53], such as *RsBCAT4*, *RsMAMa*, *RsMAMb RsIPMDH1*, *RsCPY79F1*, *RsSUR1*, *RsUTG74C1*, and *RsGSL-OH.* However, *RsGSTF9*, *RsGGP1*, *RsST5b*, and *RsAPK1* related to GSL biosynthesis had different expression levels and expression patterns between our study and Kang’s result. This may result from the difference in the genetic background of different radish genotypes. We found that several key genes (*RsBCAT4*, *RsMAM1(2)*, *RsMYB28*, and *RsGSL-OH*) of GSLs biosynthesis were closely co-expressed. The expression levels of *BCAT4* and *MAM1*, which belong to gene families encoding proteins involved in Leu biosynthesis, exhibited an extraordinarily strong correlation, which indicates that a close evolutionary relationship may exist between the pathways of GSLs chain elongation and Leu formation [19]. In addition, *RsIPMDH1* is a key gene of side chain extension in GSLs synthesis and is closely co-expressed with *RsMAM1(2)* (Figure 5). *MAM1* catalyzed the condensations in the first three elongation cycles, which were responsible for the C3/C4 chain length variation in Met-derived glucosinolates in Arabidopsis [21]. In the cabbage transgenic line M1-1, the accumulation of both GNA (C4 GSLs) and GBN (C5 GSLs) was 3.5- and 2-fold higher than that in the control, respectively [64]. The two most important GSLs in radish fleshy taproots are both 4C GSLs; therefore, we speculated that side chain extension in GSLs synthesis could be catalyzed by *RsMAM1(2)* and that *RsBCAT4* is probably the key mechanism that controls the GSLs content.

Our results also show that *RsCYP79F1*, *RsGSTF9*, *RsGGP1*, *RsSUR1*, *RsUGT74C1*, *RsST5b*, and *RsAPK1* are the key genes in the biosynthesis and accumulation of GSLs in radish fleshy taproots (Figure 3B). Genetic and transgenic approaches have been used to confirm that the biosynthesis of 3-carbon (3C) GSLs can be regulated by *CYP79F1* in *B. juncea* [65]. However, *GSL-PRO* is a probable candidate gene responsible for 3C GSLs biosynthesis in *A. thaliana*, *Brassica napus*, and *B. oleracea* [66,67]. These core-structure genes were significantly co-expressed with *RsMAM1* (Figure 5). These genes may play indispensable roles in the accumulation of 4C GSLs in radish fleshy taproots. The synergistic expression of these key genes also indicates their important roles in the GSLs synthesis process.

### 3.4. Key Genes of Indole GSLs Biosynthesis in Radish Fleshy Taproots

A very close co-expression relationship exists between *RsBCAT4* and *RsGSTF9* (Figure 5), which are responsible for the key steps of transferring sulfur during the synthesis of indole-GSLs [68]. Their expression and possible interaction may regulate the content of indole GSLs in radish fleshy taproots.

In conclusion, a comparative metabolome and transcriptome combined analysis effectively assessed the differences in GSLs profiles and gene expression between different radish cultivars with high and low GSLs content and characterized the key genes involved in radish GSLs biosynthesis and accumulation. Specifically, the key role of *RsMYB28* in the accumulation of aliphatic GSLs in radish fleshy taproots was clarified by identification of DEGs and gene co-expression analysis, and the possible regulatory relationship among *RsMYB28*, *RsSUR1*, and other 11 DEGs related to GSLs biosynthesis was also predicted. The possible molecular mechanism of 4C GSLs accumulation in radish fleshy taproots was also explored. These provide new insights into a comprehensive understanding of the mechanism of GSLs biosynthesis and for possible genetic improvement in GSLs accumulation in radish fleshy taproots. Of course, in order to confirm and apply our results, further studies are needed, such as fine mapping, cloning, and transgenic function verification of crucial genes, especially *RsMYB28*, for constructing a usable and effective regulatory network of GRH synthesis in radish genome design breeding and also in synthetic biology research to produce more beneficial GSLs for promoting human health in the future.

## 4. Materials and Methods

### 4.1. Plant Material, Cultivation, and Sampling

Based on our preliminary identification of the GSLs content in the radish taproots, three inbred lines—P16Q-17-1 (HIGH1), P16Q-23-1 (HIGH2), and P16Q-24-1 (LOW1)—with significant differences in GSLs content were selected for this study. ‘P16Q-17-1’ was a radish advanced inbred line coming from a local variety ‘Dahongpao’ in Beijing, China, and had a larger elliptical fleshy taproot with red peel and white flesh. The advanced inbred line ‘P16Q-24-1’ was bred from a local variety ‘Qiaotouqing’ in Jilin province, China, and had a long conical fleshy taproot with green peel and light green flesh. The advanced inbred line ‘P16Q-23-1’ was developed from the Japanese radish variety ‘Xiaqiumeinong’. It had a long cylindrical fleshy taproot with white peel and white flesh. A randomized block design was performed for three varieties with three replicates and fifteen plants for each replicate for each variety on the farm at the Institute of Vegetables and Flowers, Chinese Academy of Agricultural Sciences, Beijing, China. The seeds were sown in the middle of August, and fleshy taproots were harvested at the end of October 2017. The agronomic techniques were the same as those used in radish field production. The highest and lowest month average temperature was about 20 °C and 30 °C in August, about 15 °C and 28 °C in September, and 10 °C and 15 °C in October on the farm. The sandy loam with deep soil layer of water content in 65–80% and well drainage was adopted. In terms of fertilizers, we created a good balance in the application of three nutrient elements (15% N, 15% *p*, 15% K) [69]. During the fleshy taproot ripening periods, the whole taproots of five normally growing plants were collected and treated to form a replicate. Three replicates of each variety were rapidly generated, frozen in liquid nitrogen, and stored at −80 °C for GSLs analysis and RNA extraction.

### 4.2. GSLs Extraction and Analysis

GSLs were extracted according to the modified method described by He et al. [70]. Two hundred milligrams of frozen-dried taproot powder were weighed and placed into a 15 mL tube. Five milliliters of 100% methanol were added to the tube, and 100 µL of glucotropaeolin (benzylGS) was then added as an internal standard. The samples were then incubated at 83 °C for 20 min in a water bath and vortexed at 4 min to 5 min intervals. The mixture was centrifuged at 3000× *g* and 4 °C for 10 min, and the supernatant was decanted into another tube and stored in an ice bath. The extraction was repeated twice from residues using the same procedure with 70% methanol, and the supernatant solution was incorporated to obtain the sample solution. The three supernatants were combined, and 2 mL of each GSLs extract was added to a mini-column filled with diethylaminoethanol (DEAE) activated with 0.02 M NaAc and desulfated by sulfatase (Helix Pomatia Type H-1, Sigma Company, New York, NY, USA). After reaction at room temperature overnight (16 h), the desulfated GSLs were eluted with 2 mL of deionized water and stored at −20 °C for high-performance liquid chromatography (HPLC) analysis.

The HPLC system included a UV detector (SPD-20A, SHIMADZU, Tsu, Japan) with a wavelength of 230 nm, a gradient elution pump (LC-20AD, SHIMADZU, Tsu, Japan), an automatic sampler (Gina 160, Gynkotek, Germany), and a LiChrospher RP-18 analysis column (2504 mm, Merck, Germany). Mobile phase A consisted of 0.1 mol/L ammonium acetate, and mobile phase B consisted of 0.1 mol/L ammonium acetate + 300 mL of methanol; gradient elution was performed for 34 min, and all GSLs were separated. The retention times of the reference substances obtained with the newly established ESI/MS method were compared to identify GSLs. Because no phenylthioside was found in the sample, this compound was used as the internal standard. The response factors are reported with reference to Moller et al. [71].

### 4.3. RNA Extraction, Library Construction, and RNA-Seq

RNA was extracted from each biological replicate of the radish fleshy taproots using the RNAprep Pure Plant Kit (TIANGEN). After characterization of the RNA purity using a Nanodrop 1000 spectrophotometer (Thermo Fisher Scientific, Waltham, MA, USA) and measurement of the RNA concentration with a Qubit R 2.0 Fluorometer (Thermo Fisher Scientific, Waltham, MA, USA), the RNA integrity was assessed with an Agilent Bioanalyzer 2100 system (Agilent Technologies Inc. Palo Alto, CA, USA). RNA samples with an integrity higher than 7.0 were selected to construct libraries. Illumina sequencing was performed at Mega Genomics Technologies Corporation (Beijing, China). After enrichment and purification with oligo(dT)-rich magnetic beads, the mRNAs were broken into short fragments, and these fragments were converted to first- and second-strand cDNA. The cDNA was purified using AMPure XP beads, and the 3′ ends of the cDNA fragments were repaired. Poly (A) was then added and ligated to adapters for selection of a template size range. The nine cDNA libraries were then enriched by PCR amplification and sequenced using an Illumina HiSeq^TM^ 2500.

After removing the reads with only adaptors and unknown nucleotides larger than 5% or those with low quality, the clean reads were filtered from the raw reads. The clean RNA-seq reads were then mapped to the radish reference genome [55] using TopHat 2.1.1 [72]. The subsequent transcripts were then assembled, and the transcript abundance was measured based on the fragments per kilobase of exon per million fragments (FPKM) values using Cufflinks 2.2.1 [73] with the default parameters.

### 4.4. Functional Annotation of Expressed Genes and Differential Expression Analysis

Gene function was annotated based on the following databases: Nr (NCBI nonredundant protein sequences), Swiss-Prot (a manually annotated and reviewed protein sequence database), GO (Gene Ontology), KOG (EuKaryotic Orthologous Groups), COG (Clusters of Orthologous Groups of proteins), Pfam (Protein family), and KEGG (Kyoto Encyclopedia of Genes and Genomes).

A differential expression analysis among each pair of varieties was performed using the FPKM values by means of DESeq [74]. Genes with significant differential expression, denoted as DEGs, were identified based on the co-occurrence of the following two criteria: absolute value of fold change (FC) ≥ 2 and false discovery rate (FDR) value ≤ 0.01 [75]. Heatmaps were generated with the TBtoools software with log2 transformed FPKM values [76].

### 4.5. Co-Expression Analysis of the Key Genes for GSLs Biosynthesis and Their Relationship with GSLs Content in Radish Fleshy Taproots

Gene co-expression analysis and gene expression–metabolite production correlation analyses were then performed using R 3.5.0. The correlations between the expression of key genes and the GSLs content were determined by Pearson’s correlation analysis based on the criteria r ≥ |0.7| and *p* ≤ 0.01. Based on the expression levels of the selected important DEGs and the contents of the predominate GSLs components, we calculated their Pearson’s coefficients (r) and their correlation significance. Then we determined the putative key genes and components in the network according to the r values at an extremely significant level and qPCR verification results. Further, we searched the existing relationships among these key genes and GSL components discovered by previous researchers in the STRING database [77] and validated their interaction relationship again. In this way, we decided what were sources and what were targets. After that, we prepared the files (Tables S6 and S7) that included the source, target, node, Pearson’s coefficient, and the types and colors of lines designated in the network. Finally, we introduced these data into Cytoscape 3.3.0 [78] software for the interaction relationship visualization.

### 4.6. Gene Expression Validation and Analysis

Total RNA was extracted using the above-described method. A total of 800 ng of total RNA was reverse transcribed to synthesize first-strand cDNA using oligo dT primers and EasyScript^®^ One-Step gDNA Removal and cDNA Synthesis SuperMix (TransGen Biotech, Beijing, China) and diluted 20-fold to serve as templates for qPCR.

Quantitative real-time PCR was performed with a StepOne™ Real-Time PCR System (Applied Biosystems, Waltham, MA, USA) and TransStart^®^ Green qPCR SuperMix (TransGen Biotech) following the manufacturer’s instructions. Primers were designed using Primer-BLAST Web (https://www.ncbi.nlm.nih.gov/tools/primer-blast/index.cgi?LINK_LOC=BlastHome (accessed on 9 September 2019)). The length of primer is generally 15–30 bp, the product size is 100–200 bp, GC content is 40–60%, and temperature (Tm) is in the range of 57–63 °C. The list of genes and primers is shown in Table S1. Three independent biological and four technical replicates were performed. The data were analyzed using StepOne™ Software v.2.0 (Applied Biosystems, Waltham, MA, USA). Tubulin was used as an internal control. The relative expression levels were estimated using the $2^{-\Delta \Delta C_{T}}$ method [79].

## Data Availability

Not applicable.

## Supplementary Materials

The following supporting information can be downloaded at: https://www.mdpi.com/article/10.3390/ijms23062953/s1.
