# Supplementary material for: A Comparative Transcriptome and Metabolome Combined Analysis Reveals the Key Genes and Their Regulatory Model Responsible for Glucoraphasatin Accumulation in Radish Fleshy Taproots
Source: Int J Mol Sci. 2022 Mar 9;23(6):2953. doi: 10.3390/ijms23062953 (PMC8949420; doi:10.3390/ijms23062953)
Supplement: Supplementary file 1 [file ijms-23-02953-s001.zip › ijms-1598105-supplementary.pdf]

Table S1 qPCR Primers as list.

Unigene	Nr annotation	Primers (5' to 3')
Rsa10033628	*MYB28*	F: CCGTGACTCACAAGCCACT<br>R: CGGGAGTATTGCTTGTCGGA
Rsa10031835	*BCAT4*	F: GGATATCCAGTTGGGTCGGG<br>R: TCAAAGTTTCACACTGGTGGC
Rsa10025187	*IPMDH1*	F: GTCCGGAAGTCATCTCCGTC<br>R: AAAGGATTCCTCCGGCAAGG
Rsa10018392	*MAM1a*	F: GCTAGTGCGAGTGACCTCAA<br>R: ACGACACCGGAAAACGGG
Rsa10019680	*MAM1b*	F: AAGTTGGTTTCCCTGCGTCT<br>R: ATCCACCTCATTTCCCACGG
Rsa10034275	*ST5b*	F: GGGGTATGGGTTCACGGAAG<br>R: TTCGCATAAACAGCAGCACG
Rsa10041726	*APK1*	F: TGTCGAACTTTGCTTCCCGA<br>R: GACCCTTTGGATCCCTAGCC
Rsa10038067	*CYP79F1*	F: TGAAGCGGACAACCTCCTTG<br>R: AGCGTAACCATAAGCCCTCG
Rsa10032310	*GSTF9*	F: TCGCGTCAGTCATGGGATTC<br>R: ACACATCGAGAACAGCCGAG
Rsa10019051	*GGP1*	F: TTCTTGGCATCTGCTTTGGC<br>R: TCACGAGGGTTATGTCTGTAAGC
Rsa10037760	*GSL-OH*	F: CGCTATTATTCTTATCACGAACGAC<br>R: GGGTCCATAAACGCTTGCAT
Rsa10038489	*UGT74C1*	F: CCGTCTGGCTTTGTGGAAGA<br>R: GAGATAGCCTCACGTGCCAA
Rsa10031178	*SUR1*	F: ACGAGAACACTGTGGCCATT<br>R: GTCGTACACTTCGTCAGCGA

Table S2 Profiles and contents of GSL in radish taproots

Cultivar	Abbreviation	GRA	GRE	GRH	4HGBS	GBS	4MOGBS	TOL (μ mol/g DW)
P16Q-24-1	LOW1	0.00±0.00	1.08±0.27	2.74±0.45	0.14±0.02	0.11±0.02	0.18±0.03	4.26±0.71
P16Q-17-1	HIGH1	0.00±0.00	5.29±1.28	31.04±5.16	1.14±0.07	0.27±0.02	0.27±0.04	38.03±5.27
P16Q-23-1	HIGH2	0.15±0.15	4.87±0.57	29.14±4.29	1.12±0.17	0.99±0.09	0.34±0.01	36.61±4.92

Table S3 Statistics of reads generated by transcriptome sequencing of nine radish taproot samples

Sample ID	Raw Reads	Q20 content (%)	Q30 content (%)	GC content (%)	Total Reads	Mapped Reads	Mapped Ratio (%)	Unique Mapped Reads	Unique Mapped Ratio (%)
HIGH1-1	47994306	98.01	95.00	47.65	47,041,274	33,265,682	70.72	31,718,504	67.43
HIGH1-2	47982672	97.96	94.94	47.38	46,901,760	33,349,417	71.1	31,573,560	67.52
HIGH1-3	47979252	97.98	94.94	47.37	46,912,550	33,048,869	70.45	31,460,034	67.06
HIGH2-1	47992628	97.97	94.92	47.61	46,988,912	31,011,460	66.00	29,530,181	62.84
HIGH2-2	48024948	98.01	95.01	47.45	47,036,894	31,065,284	66.04	29,656,005	63.05
HIGH2-3	47997740	97.86	94.70	47.65	47,046,432	31,005,222	65.90	29,453,883	62.61
LOW1-1	45668694	97.80	94.47	47.60	44,574,590	31,582,144	70.85	29,899,709	67.08
LOW1-2	48025106	97.91	94.69	47.58	46,712,656	33,257,097	71.20	31,757,741	67.99
LOW1-3	47967424	97.99	94.85	47.51	46,509,310	32,740,986	70.40	31,226,390	67.14

Table S4 Percentages of mapped reads distributing in different sites (Exon, Intron and Intergenic).

Type	HIGH1-1	HIGH1-2	HIGH1-3	HIGH2-1	HIGH2-2	HIGH2-3	LOW1-1	LOW1-2	LOW1-3
Exon	0.817621691	0.805917012	0.802312791	0.824488277	0.818773564	0.827503679	0.809832658	0.807111418	0.801119296
Intron	0.060660974	0.063051894	0.064959438	0.057881856	0.060138571	0.057507123	0.061090419	0.063008906	0.06482301
Intergenic	0.121717335	0.131031095	0.132727772	0.117629867	0.121087865	0.114989198	0.129076923	0.129879676	0.134057694

Table S5 Number of transcripts identified in the radish fleshy taproot of each variety.

	0.5 ≤ FPKM<5	5≤FPKM<100	100≤FPKM
HIGH1	6823	14842	2238
HIGH2	6858	14321	2343
LOW1	7148	14768	2161

Table S6 Basic data of the network.

Source node	Target node	Pearson's coefficient	a/b	G/B/R
RsMAM1a	RsGGP1	0.84	a	B
RsMAM1b	RsUGT74C1	0.83	a	B
RsMAM1b	RsAPK1	0.81	a	B
RsMAM1b	RsSUR1	0.83	a	B
RsMAM1b	RsBCAT4	0.85	a	B
RsGSL-OH	RsCYP79F1	0.81	a	B
RsMYB28	RsSUR1	0.81	a	R
RsMYB28	GRH	0.81	a	G
RsMAM1a	RsMAM1b	0.96	b	B
RsMAM1a	RsUGT74C1	0.93	b	B
RsMAM1a	RsST5b	0.92	b	B
RsMAM1a	RsAPK1	0.87	b	B
RsMAM1a	RsSUR1	0.86	b	B
RsMAM1a	RsIPMDH1	0.97	b	B
RsMAM1a	RsCYP79F1	0.98	b	B
RsMAM1b	RsST5b	0.87	b	B
RsMAM1b	RsIPMDH1	0.91	b	B
RsMAM1b	RsCYP79F1	0.93	b	B
RsGGP1	RsGSL-OH	0.95	b	B
RsGGP1	RsUGT74C1	0.91	b	B
RsGGP1	RsST5b	0.96	b	B
RsGGP1	RsAPK1	0.97	b	B
RsGGP1	RsSUR1	0.99	b	B
RsGGP1	RsIPMDH1	0.91	b	B
RsGGP1	RsCYP79F1	0.87	b	B
RsGSL-OH	RsUGT74C1	0.89	b	B
RsGSL-OH	RsST5b	0.92	b	B
RsGSL-OH	RsAPK1	0.95	b	B
RsGSL-OH	RsSUR1	0.92	b	B
RsGSL-OH	RsIPMDH1	0.85	b	B
RsUGT74C1	RsST5b	0.96	b	B
RsUGT74C1	RsAPK1	0.94	b	B
RsUGT74C1	RsSUR1	0.93	b	B
RsUGT74C1	RsIPMDH1	0.97	b	B
RsUGT74C1	RsCYP79F1	0.97	b	B
RsST5b	RsAPK1	0.95	b	B
RsST5b	RsSUR1	0.97	b	B
RsST5b	RsIPMDH1	0.97	b	B
RsST5b	RsCYP79F1	0.95	b	B
RsAPK1	RsSUR1	0.96	b	B
RsAPK1	RsIPMDH1	0.93	b	B
RsAPK1	RsCYP79F1	0.91	b	B

RsSUR1	RsIPMDH1	0.93	b	B
RsSUR1	RsCYP79F1	0.90	b	B
RsIPMDH1	RsCYP79F1	0.99	b	B
GRE	GRH	0.92	b	G
RsBCAT4	RsGSTF9	0.92	b	B

Note: a/b: the lines in network are shown as solid (a) or dashed (b). G/B/R: it is the abbreviation of Green/Blue/Red.

Table S7 Node information of network.

Node	Color
RsMAM1a	blue
RsMAM1b	blue
RsGGP1	blue
RsGSL-OH	blue
RsMYB28	red
RsUGT74C1	blue
RsST5b	blue
RsAPK1	blue
RsSUR1	blue
RsIPMDH1	blue
RsCYP79F1	blue
RsBCAT4	blue
GRH	green
GRE	green
RsGSTF9	blue
